# A Novel Non-invasive Mouthpiece Model for Evaluating Oral Hypofunction: Focus on Mastication and Stomatognathic Dynamics

**DOI:** 10.7759/cureus.82067

**Published:** 2025-04-11

**Authors:** Mika Kikuchi, Ikuo Yonemitsu, Takashi Ono, Koji Saisho, Yuka Tanaka-Takemura, Mayuka Watanabe, Hiroshi Takemura, Kohei Soga, Kazuhiro Suga, Motohiro Uo

**Affiliations:** 1 Graduate School of Medical and Dental Sciences, Orthodontic Science, Institute of Science Tokyo, Tokyo, JPN; 2 Faculty of Science and Technology, Mechanical and Aerospace Engineering, Tokyo University of Science, Chiba, JPN; 3 Faculty of Advanced Engineering, Medical and Robotic Engineering Design, Tokyo University of Science, Tokyo, JPN; 4 Department of Mechanical Engineering, Faculty of Engineering, Kogakuin University, Tokyo, JPN; 5 Graduate School of Medical and Dental Sciences, Advanced Biomaterials, Institute of Science Tokyo, Tokyo, JPN

**Keywords:** bite force, canine guidance, malocclusion, mastication ability, mouthpiece, occlusal contact areas, oral hypofunction, stomatognathic system

## Abstract

Background/Objectives: Studies have examined the mechanisms of oral hypofunction by investigating the relationship between the number of remaining teeth, bite force, mastication ability, and lateral induction factors influencing chewing pathways. However, many previous studies have not consistently assessed the same individuals across all these factors, leaving key contributing factors unaddressed. This study aimed to develop a reliable oral hypofunction model using non-invasive mouthpieces. We hypothesized that it is possible to reproduce a model of oral hypofunction in healthy individuals using a mouthpiece by modifying occlusal contact areas and lateral induction factors.

Methods: A total of 10 healthy adults (five men and five women; mean age: 28.4 ± 1.5 years old) with normal occlusion and no stomatognathic abnormalities participated in this study. Experiments were conducted under four conditions: (1) no mouthpiece; (2) a mouthpiece with large occlusal contact areas and canine guidance; (3) a mouthpiece with large occlusal contact areas but no canine guidance; and (4) a mouthpiece with small occlusal contact areas and no canine guidance. The outcomes assessed were maximum bite force, occlusal contact area, mastication ability, and salivation.

Results: A strong positive correlation was identified between occlusal contact area and maximum bite force (p < 0.001). Furthermore, occlusal contact was positively correlated with mastication ability (p < 0.001). The glucose concentration of the filtered solution decreased significantly from Condition I (mean: 203.3 (SD = 42.7) mg/dl) to Condition IV (mean: 90.2 (SD = 14.2) mg/dl) (Figure 9). The presence of lateral induction factors, such as canine guidance, demonstrated a significant influence on these outcomes (p < 0.05).

Conclusions: The findings align with those of previous studies and validate the use of this mouthpiece model for simulating oral hypofunction. This model, adaptable for use in healthy individuals, supports larger-scale studies under controlled conditions to better elucidate the mechanisms underlying oral hypofunction.

## Introduction

Mastication is a fundamental component of the stomatognathic system and is crucial in sustaining overall health and life [1]. Malocclusion negatively impacts masticatory function [2,3], and its relationship with factors such as the number of remaining teeth [4-6], bite force, and chewing ability [4,7,8], as well as occlusal guidance influencing mandibular trajectories, have been extensively studied [9]. However, many of these studies have been limited to group comparisons between individuals with healthy oral function and those experiencing hypofunction, often neglecting intra-individual variability. Consequently, the potential interplay between additional factors, such as oral hygiene [10], dryness [11], bite force, and tongue-lip movement remains insufficiently understood [12]. Furthermore, it is important to note that oral hypofunction does not only affect the mechanical aspects of mastication but also has far-reaching implications for systemic health, including nutritional deficiencies and an increased risk of frailty in the elderly population [13].

We hypothesized that it is possible to reproduce a model of oral hypofunction in healthy individuals using a mouthpiece by modifying individual factors. In this study, by only using a mouthpiece the conditions of the occlusal contact state can be changed and examined its impact on other conditions. While previous studies [1-4] have manipulated occlusal factors, such as occlusal contact areas and canine guidance, using intraoral appliances, to our knowledge, no study has specifically compared different occlusal conditions while maintaining a given mouthpiece thickness. The novelty of our study lies in ensuring that factors other than occlusion remain consistent, allowing for precise evaluation of occlusal effects. This approach allows for an analysis of functional declines under controlled conditions, offering insights into the nuanced interplay of multiple factors that influence oral function.

Diagnostic criteria for oral hypofunction have been developed to assess oral functional decline in older individuals [14,15]. Oral hypofunction refers to a condition in which the function of the stomatognatic system has decreased due to aging, disease, tooth decay, periodontal disease, side effects of drugs, lack of nutrition, etc. However, no specific methods have been proposed to prevent and improve oral dysfunction [13,16,17]. This condition encompasses a complex reduction in chewing, swallowing, vocalization, and salivation, which, if untreated, can lead to malnutrition, frailty, and systemic health deterioration. Effective prevention and management necessitate a deeper understanding of the mechanisms underlying masticatory dysfunction [13,16,17]. Although previous studies [18-20] have highlighted the importance of early interventions during childhood to support normal growth and development, specific preventive methods remain undefined. To address these gaps, this study aimed to develop a noninvasive oral hypofunction model using a mouthpiece. While oral hypofunction encompasses various functional declines, including swallowing, salivation, and articulation, this study specifically focuses on the mastication.

This article was previously presented as a meeting abstract at the 83rd Annual Meeting of the Japanese Orthodontic Society held from October 29 to 31, 2024, and posted to the preprint server at Preprints.org on January 21, 2025 (DOI: 10.20944/preprints202501.1499.v1).

## Materials and methods

Study design

This cross-sectional study adhered to the ethical principles outlined in the Declaration of Helsinki and was approved by the Ethics Review Committee of the Institute of Science, Tokyo, Japan (approval number: S2024-018). Participants received detailed verbal explanations regarding the study's objectives, methods, safety measures, and potential risks and provided written informed consent prior to participation.

The experiment was conducted in Orthodontic Science, Institute of Science Tokyo from September 5, 2023, to December 20, 2024. The research was carried out and the examined variables were measured by M.K.

Participants

The inclusion criteria for participants were as follows: healthy adults aged 26-32 years; normal occlusion with no abnormalities in the stomatognathic system; a minimum of 24 functional teeth; absence of prosthetic teeth, crossbite, scissors bite, or open bite; no history of masticatory disorders or muscular diseases; no cognitive impairments; ability to chew gummy jelly and no known gelatin allergies.

Exclusion criteria were congenital abnormalities (e.g., cleft lip and palate); Severe mandibular or nasal bone curvature; Inability to evaluate mandibular shape due to facial hair, including beards and sideburns; A BMI outside the range of 18.5-25, as defined by Japan Obesity Society standards; Withdrawal of consent at any stage of the study.

Prior to this study, the sample size was determined based on a power analysis, using EZR (Saitama Medical Center, Jicji Medical University, Saitama, Japan), which is a graphical user interface for R (The R Foundation for Statistical Computing, Vienna, Austria). Assuming an alpha level of 0.05 and a power of 0.80, the required sample size was estimated to be 7 participants. The data used for this power analysis were derived from our own unpublished pilot study. In this pilot study, we measured the masticatory function as the primary outcome variable, with an effect size of 0.6 (medium effect), and a standard deviation of 1.2. To account for potential dropouts, we recruited 10 participants [21].

Intervention

**Figure 1:**
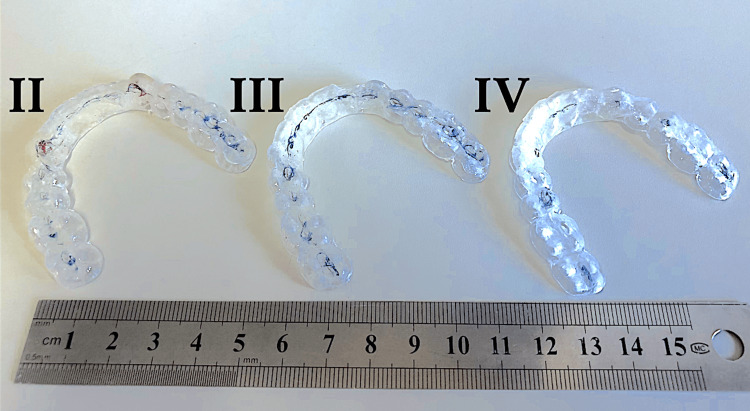
Mouthpieces The intercuspal position is marked in blue, while the lateral guide elements are marked in red using articulating paper. Experiments were conducted under four conditions: (I) no mouthpiece; (II) a mouthpiece with large occlusal contact areas and canine guidance; (III) a mouthpiece with large occlusal contact areas but no canine guidance; and (IV) a mouthpiece with small occlusal contact areas and no canine guidance. Image taken by Mika Kikuchi, used with permission from JM Ortho.

The experiments were conducted under four conditions (Figure 1): I: No mouthpiece; II: A mouthpiece with large occlusal contact areas and canine guidance; III: A mouthpiece with large occlusal contact areas but without canine guidance; IV: A mouthpiece with small occlusal contact areas without canine guidance.

**Figure 2:**
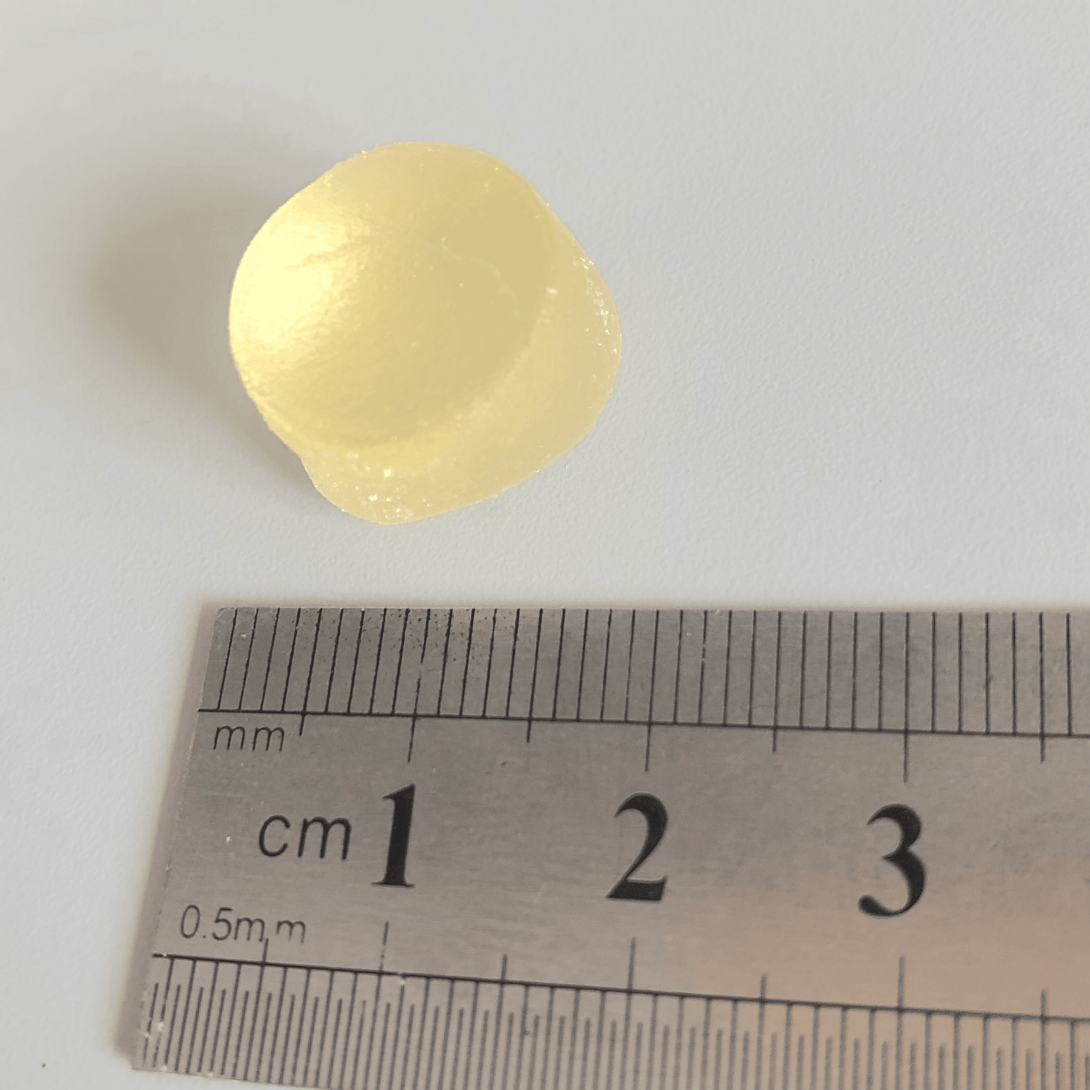
Glucose-containing gummy jelly (GLUCOLUMN; GC Corp., Tokyo, Japan). Image taken by Mika Kikuchi, used with permission from GC Corp.

Glucose-containing gummy jelly (Figure 2; GLUCOLUMN; GC Corp., Tokyo, Japan; [22] diameter: 14 mm; height: 8 mm; weight: approximately 2 g) was masticated on the habitual chewing side for 20 seconds under each condition. After mastication, subjects rinsed their mouths with 10 ml of water and expectorated the jelly into a filtration device. Each condition was tested in 3 sets, with a 15-minute break between conditions and a 3-minute break between sets to minimize fatigue.

Mouthpieces were fabricated using a thermoplastic resin (NEWIMPRELON S PD; JM Ortho, Tokyo, Japan; thickness = 1.5 mm) and adjusted with a polymerization resin (UNIFAST III; GC Corp., Tokyo, Japan) to ensure consistent bite height across conditions. Adjustments were made to prevent discomfort or interference with lip and tongue movements. All interventions were conducted between 13:00 and 17:00 on the same day, and participants were not required to fast prior to the study.

Outcomes

The outcomes were evaluated under each condition. Maximum bite force, occlusal contact areas, masticatory ability, and salivation were measured. To minimize fatigue, a 15-minute break between each condition and a 3-minute break between each experiment were given.

Maximum Bite Force and Occlusal Contact Areas

**Figure 3:**
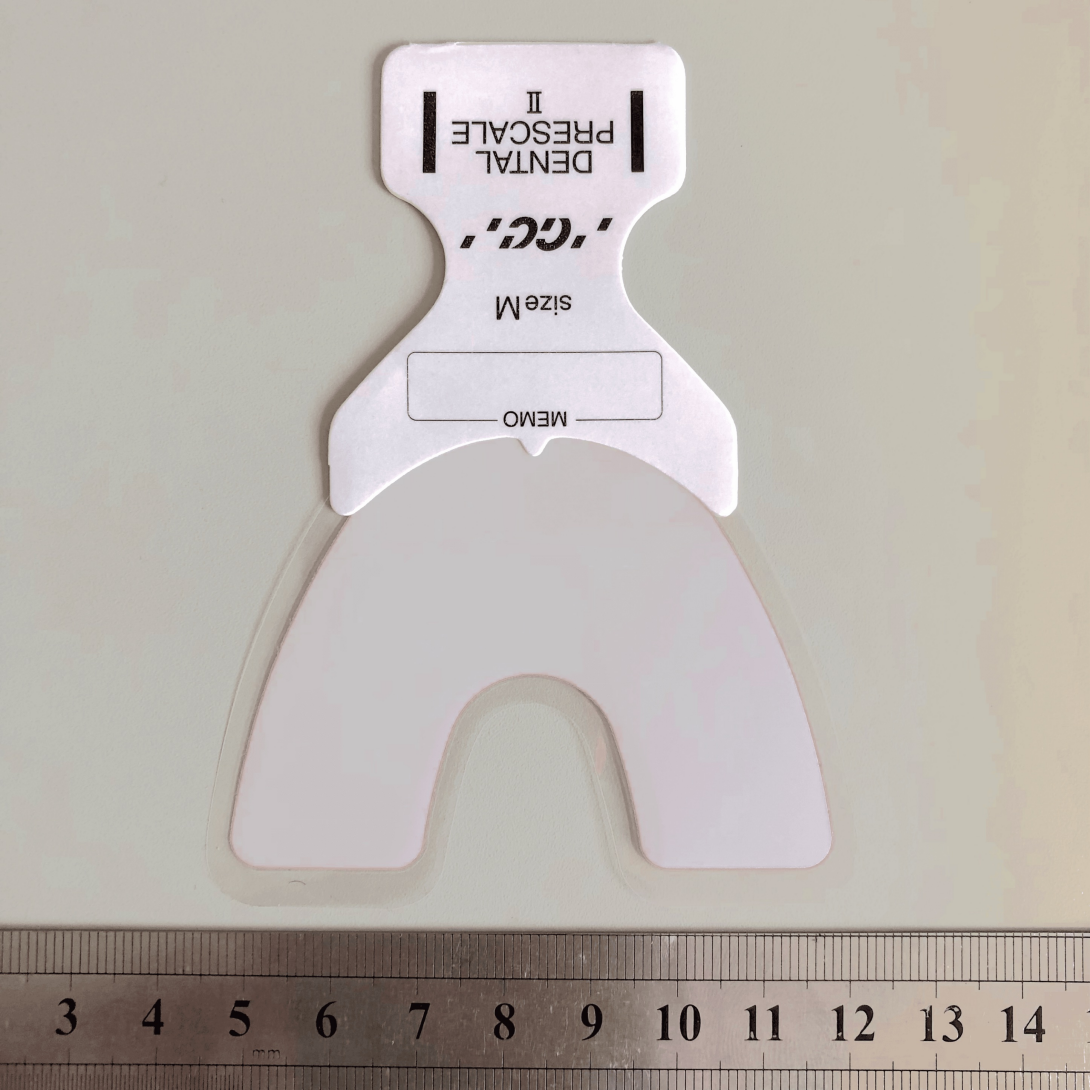
Device for measuring maximum bite force and occlusal contact area (Dental Prescale II, GC Corp., Tokyo, Japan). Image taken by Mika Kikuchi, used with permission from GC Corp.

Maximum occlusal force and contact areas were measured using a pressure-sensitive film (Figure 3; Dental Prescale II; GC Corp., Tokyo, Japan). The film was inserted into the oral cavity to ensure complete coverage of the dentition, and participants were instructed to bite the film for three seconds. The occlusal state was analyzed using occlusal force analysis software (Bite Force Analyzer; GC Corp., Tokyo, Japan) [23,24].

Chewing Ability

**Figure 4:**
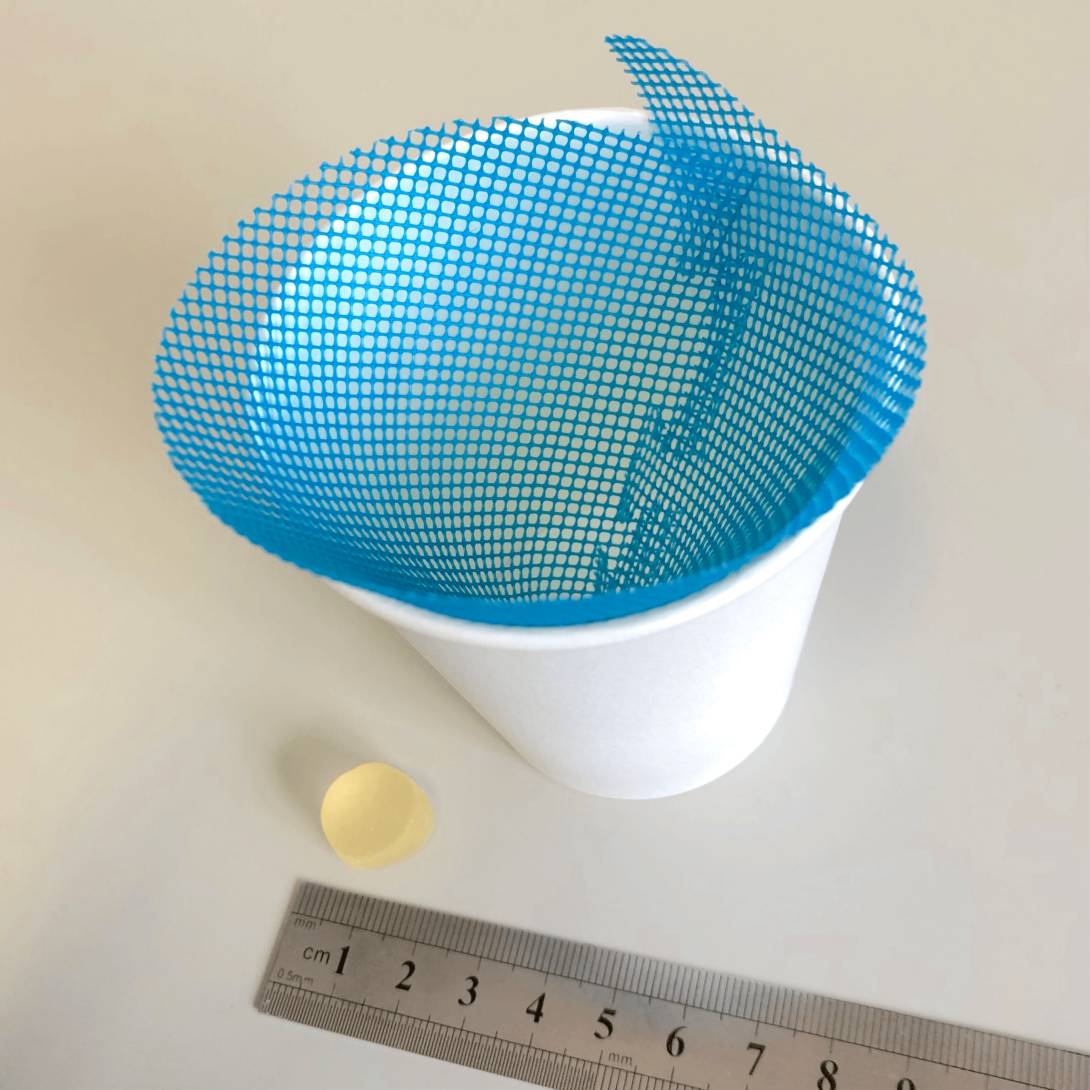
Filtration device for glucose concentration, consisting of a mesh filter and a paper cup. Image taken by Mika Kikuchi, used with permission from GC Corp.

Glucose-containing gummy jelly was used to evaluate chewing ability. Participants masticated the gummy jelly on their habitual chewing side for 20 seconds without swallowing. After mastication, 10 ml of water was added to the mouth and gently rinsed, and the combined mixture of masticated gummy jelly and water was expectorated onto a filter (Figure 4). The filtrate concentration was then analyzed using a glucose sensor (GLUCOSENSOR; GC Corp., Tokyo, Japan) [22]. Additionally, the gummy jelly was maintained at room temperature to prevent changes in hardness due to temperature fluctuations.

Salivation

The amount of saliva was measured by weighing the expectorated contents after chewing, and the weight of the gummy jelly was subtracted to calculate the net saliva production. Since this study was a comparative experiment conducted with the same subjects, it was assumed that baseline salivary flow at rest did not vary significantly. However, because the method used to measure chewing ability in this study depended on the glucose concentration, the saliva volume was also measured to ensure accurate interpretation [25]. This was essential because if a large volume of saliva was secreted, it could dilute the glucose solution, lowering its concentration. This could lead to a misinterpretation of the results, where a decrease in mastication ability might mistakenly be attributed to the condition itself, rather than the dilution of the glucose solution caused by excessive saliva. By measuring saliva volume, we could rule out this possibility and ensure more accurate data interpretation.

Data analysis

All statistical analyses were performed using EZR (Saitama Medical Center, Jichi Medical University, Saitama, Japan), a modified version of R Commander (version 1.6-3) designed to incorporate statistical functions frequently used in biostatistics.

Initially, raw data were checked for normality using the Shapiro-Wilk test and for homogeneity of variance using Levene’s test. For normally distributed data, parametric tests such as independent t-tests and one-way ANOVA were applied. When the data did not meet normality assumptions, non-parametric alternatives such as the Mann-Whitney U test and Kruskal-Wallis test were used.

Correlation analysis was conducted using Pearson’s or Spearman’s correlation coefficients, depending on data distribution. All P-values were two-sided, and statistical significance was set at p < 0.05.

## Results

In this study, we included 10 participants, whose demographic characteristics are summarized in Table 1. The mean age of the participants was 28.4 years, with a gender distribution of five males and five females. Additional demographic data, such as BMI and number of functional teeth, are also provided.

**Table 1:** Demographic Data The Table 1 summarizes the demographic data of the participants involved in the study.

Gender	n	Age (Mean ± SD)	Age Range	BMI (Mean ± SD)	Number of Functional Teeth
Female	5	28.0 ± 1.41	26 - 30	21.0 ± 1.8	26.8 ± 1.6
Male	5	28.8 ± 1.79	28 - 32	20.9 ± 1.5	26.4 ±2.0
Total	10	28.4 ± 1.51	26 - 32	20.9 ± 1.7	26.6 ± 1.8

Salivation measurement

**Figure 5:**
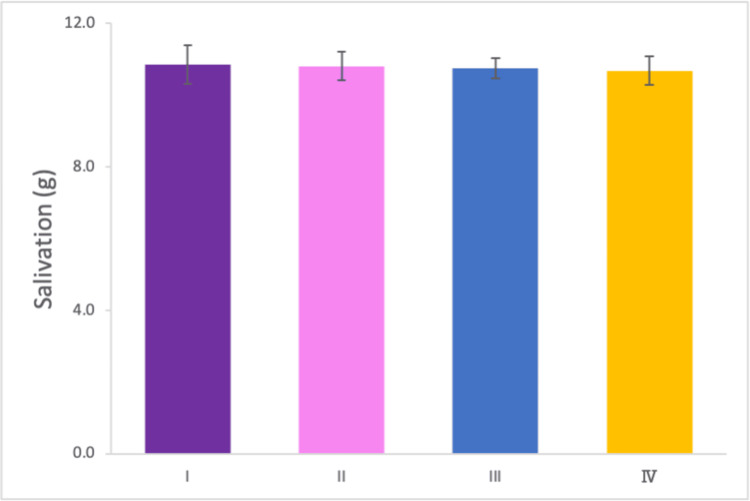
Weight of the glucose solution containing 10 ml of water. No significant differences in the weight of the glucose solution across the four conditions. Error bar represents standard deviation. Experiments were conducted under four conditions: (I) no mouthpiece; (II) a mouthpiece with large occlusal contact areas and canine guidance; (III) a mouthpiece with large occlusal contact areas but no canine guidance; and (IV) a mouthpiece with small occlusal contact areas and no canine guidance.

The weight of the filtrate obtained from 10 ml of rinsing water after chewing the gummy jelly was measured, representing the weight of the glucose solution mixed with saliva under four experimental conditions. No significant difference was observed in the solution weight across conditions, indicating that neither the occlusal state nor the use of a mouthpiece influenced saliva secretion (Figure 5).

Relationship between occlusal contact areas and maximum bite force

Measurement of Occlusal Contact Areas

**Figure 6:**
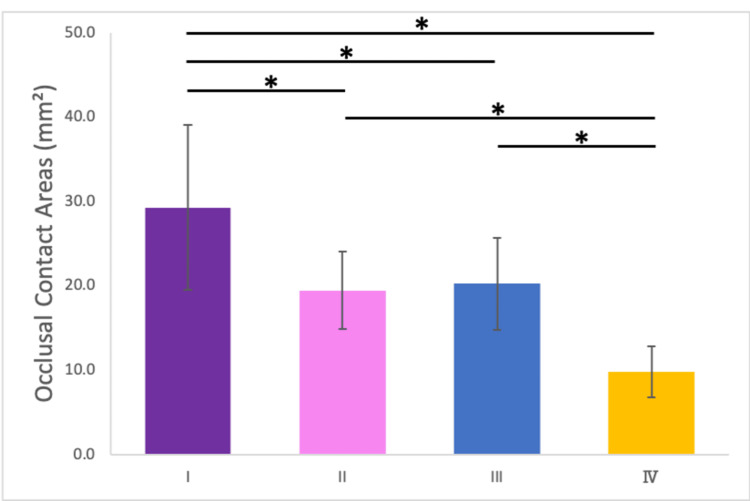
Occlusal contact areas under the four conditions. Error bar represents standard deviation. *: p < 0.05. Experiments were conducted under four conditions: (I) no mouthpiece; (II) a mouthpiece with large occlusal contact areas and canine guidance; (III) a mouthpiece with large occlusal contact areas but no canine guidance; and (IV) a mouthpiece with small occlusal contact areas and no canine guidance.

The occlusal contact areas were significantly larger under Condition I (29.3 ± 9.8 [mean ± standard deviation (SD) mm2] compared to the other conditions. No significant differences were observed between Conditions II (19.5 ± 4.6 mm2) and III (20.2 ± 5.4 mm2), while Condition IV (9.8 ± 3.1 mm2) exhibited the smallest occlusal contact area (Figure 6).

Relationship Between Occlusal Contact Areas and Maximum Bite Force

**Figure 7:**
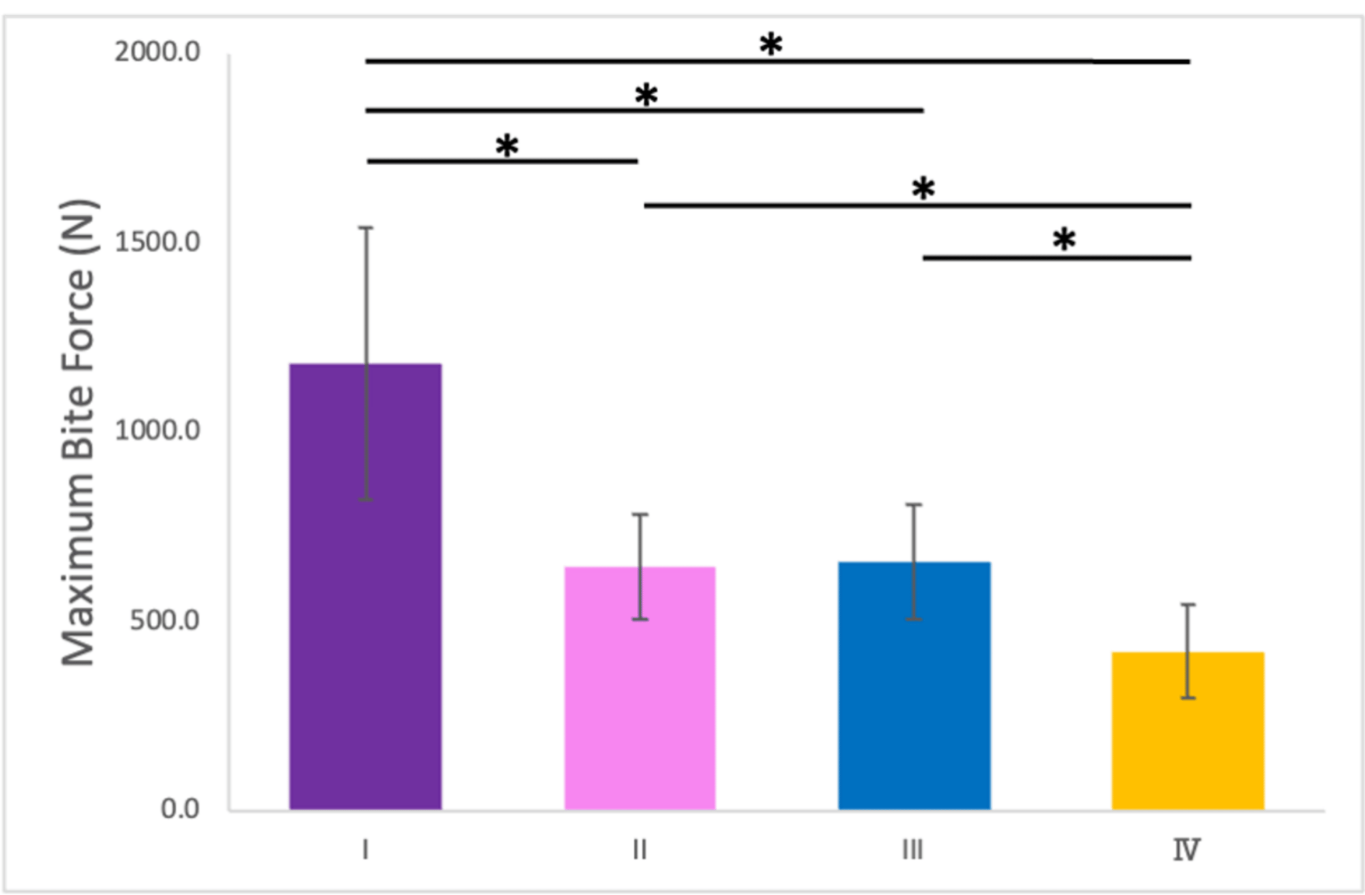
Maximum bite force under the four conditions. Error bar represents standard deviation. *: p < 0.05 Experiments were conducted under four conditions: (I) no mouthpiece; (II) a mouthpiece with large occlusal contact areas and canine guidance; (III) a mouthpiece with large occlusal contact areas but no canine guidance; and (IV) a mouthpiece with small occlusal contact areas and no canine guidance.

The maximum bite forces measured under the four conditions are shown in Figure 7. The average maximum bite force for Japanese males is approximately 800-1,200 N, while for females, it ranges from 500-800 N [24]. Conditions II (644.5 ± 139.4 N) and III (655.0 ± 151.1 N) showed no significant differences in bite force. However, the bite force was significantly reduced in Condition IV (419.6 ± 122.9 N) compared with Conditions II and III.

**Figure 8:**
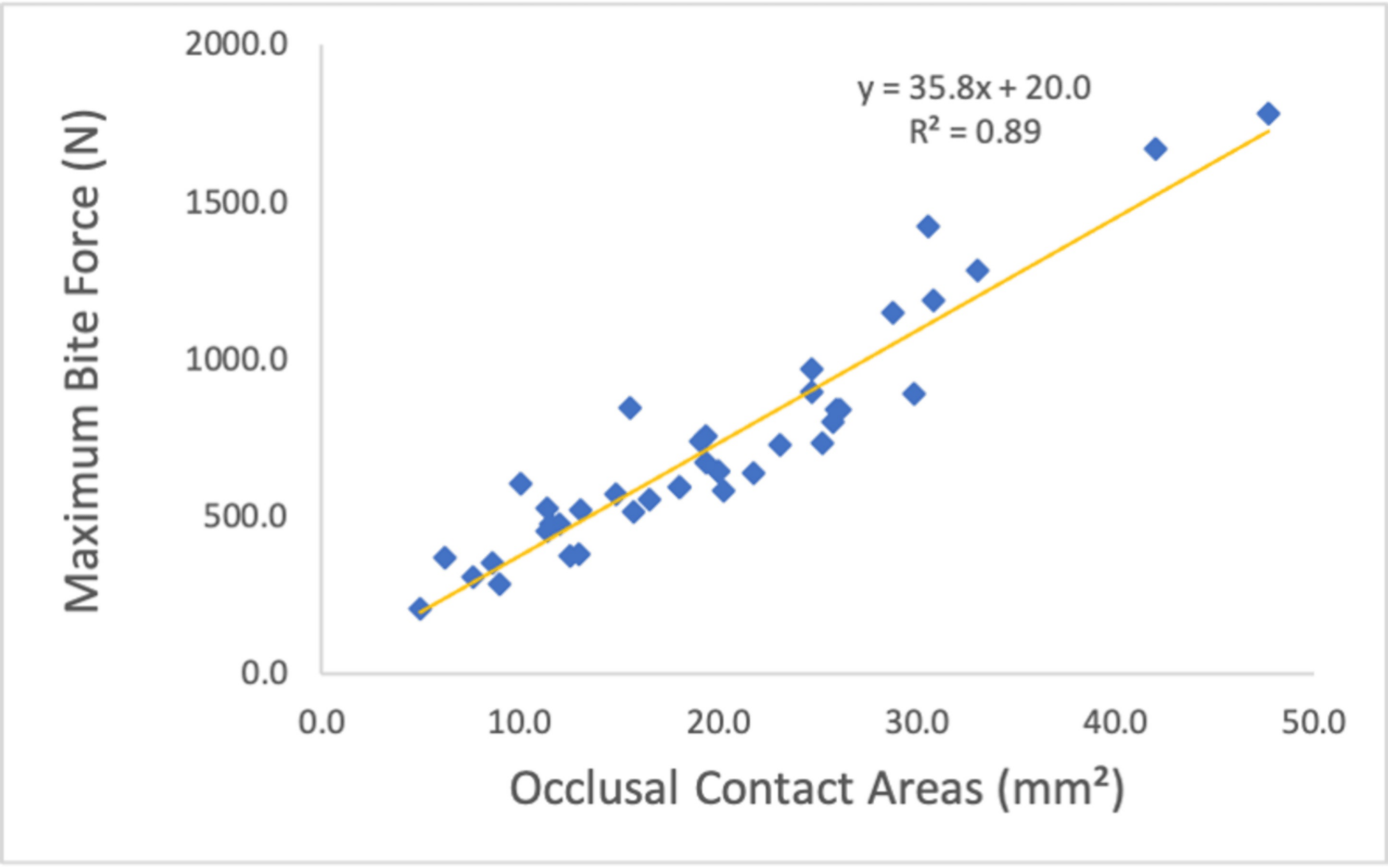
Relationship between occlusal contact areas and maximum bite force.

The maximum bite force mirrored the pattern observed for occlusal contact areas, with a significant positive correlation between the two variables (r = 0.943, p < 0.001; Figure 8).

Relationship Between Occlusal Contact Areas and Glucose Concentration

Measurement of Glucose Concentration

**Figure 9:**
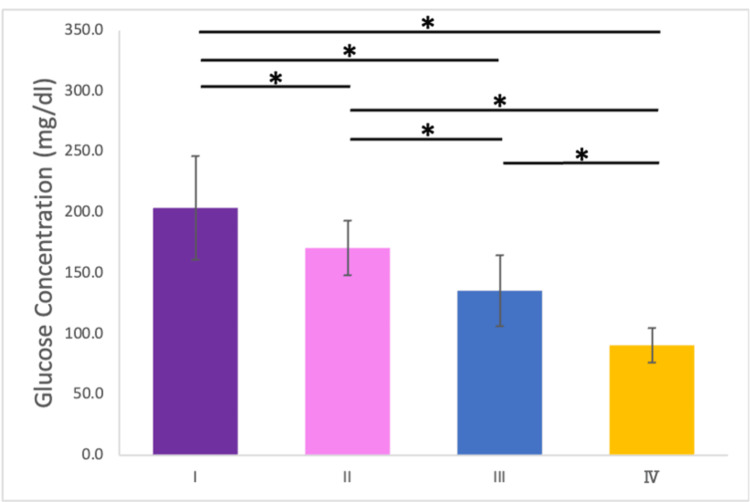
Glucose concentration across the four conditions. Error bar represents standard deviation. *: p < 0.05. Experiments were conducted under four conditions: (I) no mouthpiece; (II) a mouthpiece with large occlusal contact areas and canine guidance; (III) a mouthpiece with large occlusal contact areas but no canine guidance; and (IV) a mouthpiece with small occlusal contact areas and no canine guidance.

The glucose concentration of the filtered solution decreased significantly from Condition I (203.3 ± 42.7 mg/dl) to Condition IV (90.2 ± 14.2 mg/dl) (Figure 9).

Relationship Between Occlusal Contact Areas and Glucose Concentration

**Figure 10:**
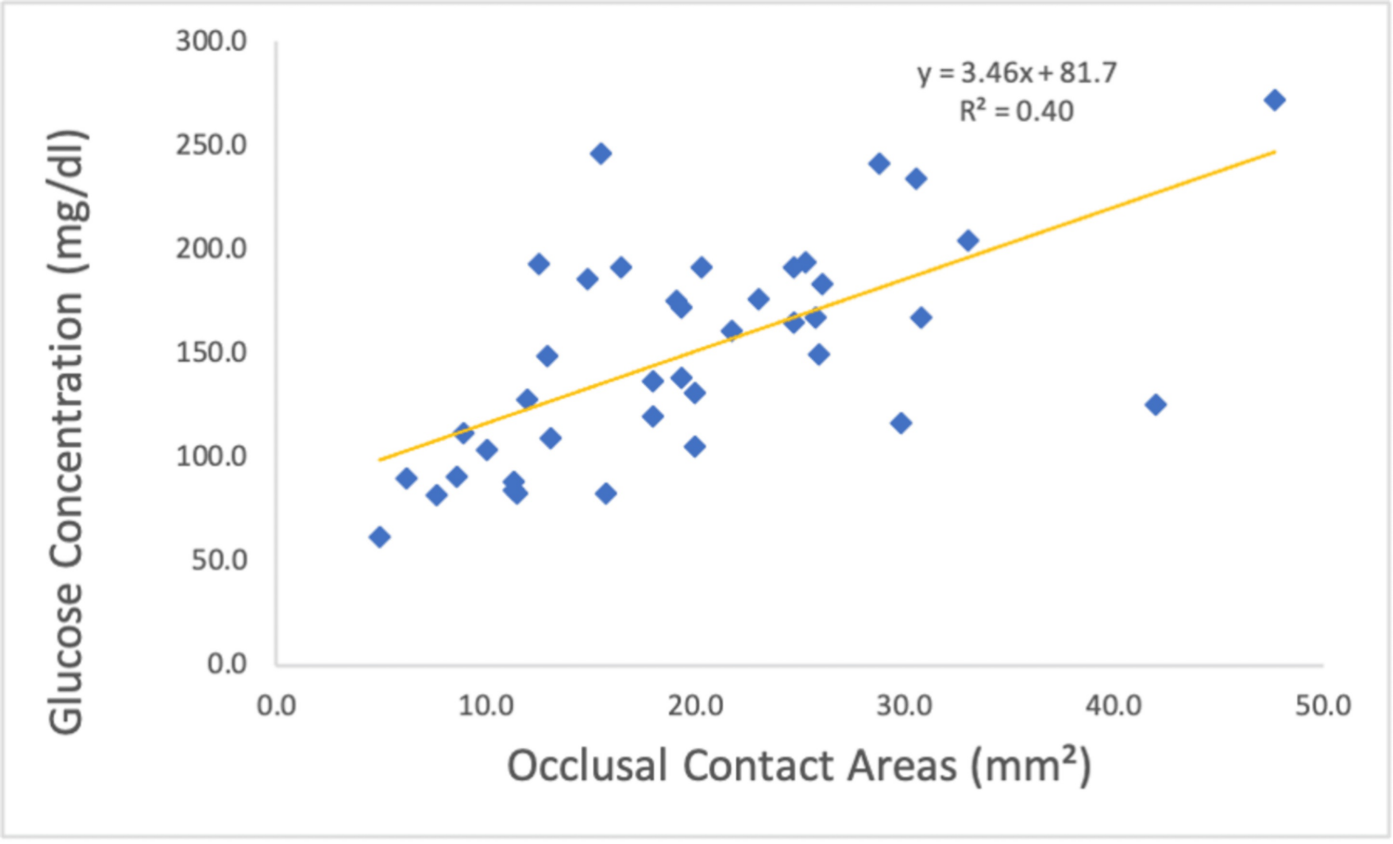
Relationship between occlusal contact areas and glucose concentration.

In contrast to the relationship between bite force and occlusal contact areas, a significant correlation was observed between occlusal contact areas and chewing ability, as indicated by glucose concentration (r = 0.630, p < 0.001; Figure 10).

## Discussion

This study aimed to establish an oral hypofunction model using a mouthpiece and evaluate its effects on salivation, occlusal contact areas, maximum bite force, and mastication ability under controlled conditions. The findings are consistent with previous research [3,4,7,25-28] and provide novel insights into the relationship between occlusal characteristics and masticatory performance.

Salivation

The absence of significant differences in salivation across conditions suggests that neither the occlusal state nor the presence of a mouthpiece significantly affects saliva secretion. This finding validates the experimental design, ensuring that variations in salivation did not confound the other measured outcomes [25]. However, it is worth noting that salivary response is influenced by various external factors, such as dietary stimuli and psychological stress, which were controlled to the best extent possible during this study. Future investigations could further explore the long-term impact of modified occlusal states on salivary flow rate and composition to understand potential chronic effects on oral health.

Occlusal contact areas and bite force

A significant positive correlation was identified between occlusal contact areas and maximum bite force. In Condition II, the occlusal contact areas were significantly smaller than those in Condition I. Under normal occlusion, the upper and lower teeth contact each other with the occlusal cusps fitting into the opposing occlusal cavities [26]. However, in Condition II, the occlusal cusps of the maxilla were not reproduced in the mouthpiece, resulting in a “point contact” occlusion. Additionally, comparisons between Condition I and the other conditions require careful interpretation. Condition I had a lower occlusal vertical dimension compared to Conditions II, III, and IV. Comparing Conditions II and III, no significant differences were observed in the occlusal contact area and occlusal force, as the only variable distinguishing these conditions was the lateral induction factor. Contrastingly, comparing Conditions II and III with Condition IV revealed a significant decrease in occlusal force, attributed to a reduction in occlusal contact area by approximately half. This finding was consistent with those of previous studies [4,7]; however, those studies may have been influenced by factors such as muscle strength, as they did not compare results within the same subjects. In this study, the significant decrease in maximum bite force observed with the reduction in occlusal contact areas can be attributed to the controlled experimental conditions, as all measurements were performed on the same subjects under identical protocols. Furthermore, differences in mandibular position can also influence sensory feedback from the periodontal tissues, which in turn modulates bite force. Indeed, Kishimoto et al. (2019) demonstrated that afferent input from the periodontal ligament activates the prefrontal cortex, leading to downregulation of occlusal force [29]. Additional research will help to investigate how other factors, such as muscle fatigue and temporomandibular joint mechanics, interact with occlusal adjustments.

Chewing ability

A significant positive correlation was observed between occlusal contact areas and chewing ability. A comparison of Conditions II and III revealed that the presence of lateral induction factors, such as canine guidance, had a substantial impact on chewing ability. A previous study [27] highlighted that lateral induction factors influence mandibular movement pathways during chewing, establishing a relationship between mandibular trajectories and chewing ability [28]. In this study, the loss of lateral induction factors altered mandibular trajectories, leading to a significant deterioration in chewing ability in Conditions III and IV. Additionally, lateral induction factors take various forms, such as group function and canine tooth induction. In this study, canine tooth induction was emphasized for its high reproducibility and minimal interference with the molars on the habitual and opposite chewing sides. However, as the presence or absence of canine tooth induction was not a criterion for subject selection, some subjects did not exhibit canine tooth induction. For these individuals, the glucose concentration in Condition II was higher than that of Condition I. Comparing Conditions III and IV under the same lateral induction factors revealed a significant decrease in chewing ability as the occlusal contact area decreased. While no previous studies have specifically examined the effects of conditions with or without lateral induction factors, previous studies [3,4] have shown that chewing ability decreases as occlusal contact areas decrease. This aligns with the findings of this study and highlights the complex interplay between occlusal factors and mandibular biomechanics. We also acknowledge that a glucose concentration of 100 mg/dl or lower is considered an indicator of impaired mastication function, which can be regarded as a gold standard in this context [30]. In Condition IV, where the glucose concentration was below this threshold, we believe that a state of impaired mastication function was effectively recreated.

Clinical implications

These findings are consistent with those of previous studies, validating the mouthpiece model as a reliable tool for simulating oral hypofunction. The mechanism of oral hypofunction has been explored in studies investigating the relationships between the number of remaining teeth, bite force, mastication ability, and lateral induction factors affecting chewing pathways. However, many of these studies have lacked intrasubject verification, leaving key influencing factors unaddressed. This model offers a noninvasive and accessible method for simulating oral hypofunction in healthy individuals, enabling controlled intrasubject comparisons. Such an approach facilitates the understanding of how occlusal variables influence mastication mechanics, providing valuable insights into the complex interactions within the stomatognathic system. Future research will be able to extend the application of this model to explore therapeutic interventions, such as customized prosthetics or orthodontic devices, and their efficacy in restoring normal occlusal function. Although long-term prognosis assessments remain challenging, this model enables the replication of post-treatment oral conditions, allowing for the preliminary evaluation and prediction of treatment effects. By simulating different occlusal environments, it may also be useful for testing compensatory strategies in individuals with existing occlusal dysfunction. Furthermore, longitudinal studies would assess the model’s utility in predicting the deterioration of oral hypofunction over time, thereby guiding preventive strategies. The simplicity and scalability of the model make it a valuable tool for future research. Furthermore, longitudinal studies would assess the model’s utility in predicting the deterioration of oral hypofunction over time, thereby guiding preventive strategies. The simplicity and scalability of the model make it a valuable tool for future research. Furthermore, its ability to reproduce various malocclusions observed in clinical practice makes it useful for studying a wide range of chewing malfunctions.

Limitations

This study has several limitations. First, the sample size was relatively small, with only 10 participants, which may limit the generalizability of the findings. Second, the study focused on a specific age group, with participants aged between 26 and 32 years, which may not fully represent other age demographics. Finally, the research was conducted over a limited period, and therefore, the long-term effects of occlusal conditions could not be assessed. These constraints should be taken into consideration when interpreting the results.

Additionally, since the presence or absence of canine tooth guidance was not a criterion for subject selection, some participants did not exhibit canine tooth guidance. Although significant differences in masticatory function were observed among Groups II, III, and IV, regardless of the participants' original occlusal state, it is important to acknowledge that the presence or absence of canine guidance may influence mandibular movement. Future studies should consider comparing groups with and without natural canine guidance to further clarify its impact.

Furthermore, adapting this model for elderly populations would be a valuable next step, as it would allow for the assessment of age-related changes in masticatory function and their implications for oral hypofunction. Finally, while this study primarily focused on short-term effects, future longitudinal studies could provide deeper insights into the progression of oral hypofunction and the long-term efficacy of therapeutic interventions. These extensions would help broaden the applicability of our findings and contribute to the development of targeted preventive and rehabilitative strategies.

## Conclusions

This study provides a descriptive analysis of relevant correlations observed within the same group, using a mouthpiece to establish an oral hypofunction model. The findings are consistent with previous research, and the model allows intrasubject comparisons under controlled conditions, enabling precise analysis of factors influencing masticatory performance. Specifically, the observed associations between occlusal contact areas, bite force, and masticatory ability align with prior studies, supporting the validity of our approach. The model was introduced as a potential framework, and its adaptability to healthy subjects and ease of use make it a promising method for advancing research into the mechanisms underlying oral hypofunction and for designing effective clinical interventions. Further studies are necessary to evaluate its applicability in clinical settings and broader research contexts.
